# 259. Long-term Transcriptomic Changes in the CD4 T cells Following Treatment of Extrapulmonary Tuberculosis

**DOI:** 10.1093/ofid/ofad500.331

**Published:** 2023-11-27

**Authors:** Seong Jin Choi, Young-Jun Kim, Jeong Seok Lee, Yunsang Choi, Song Mi Moon, Nak-Hyun Kim, Kyong-Ho Song, Eu Suk Kim, Chang Kyung Kang, Pyoeng Gyun Choe, Wan Beom Park, Nam Joong Kim, Myoung-don Oh, Eui-Cheol Shin, Hong Bin Kim

**Affiliations:** Seoul National University Bundang Hospital, Seoungnam-si, Kyonggi-do, Republic of Korea; Wonkwang University HospitalSpreading into the world, Iksan, Cholla-bukto, Republic of Korea; Korea Advanced Institute of Science and Technology (KAIST), Daejeon, Taejon-jikhalsi, Republic of Korea; Seoul National University Bundang Hospital, Seoungnam-si, Kyonggi-do, Republic of Korea; Seoul National University Bundang Hospital, Seoungnam-si, Kyonggi-do, Republic of Korea; Seoul National University Bundang Hospital, Seoungnam-si, Kyonggi-do, Republic of Korea; Seoul National University Bundang Hospital, Seoungnam-si, Kyonggi-do, Republic of Korea; Seoul National University Bundang Hospital, Seoungnam-si, Kyonggi-do, Republic of Korea; Seoul National University College of Medicine, Seoul, Seoul-t'ukpyolsi, Republic of Korea; Seoul National University College of Medicine, Seoul, Seoul-t'ukpyolsi, Republic of Korea; Seoul National University College of Medicine, Seoul, Seoul-t'ukpyolsi, Republic of Korea; Seoul National University College of Medicine, Seoul, Seoul-t'ukpyolsi, Republic of Korea; Department of Internal Medicine, Seoul National University College of Medicine, Seoul, Korea, Seoul, Seoul-t'ukpyolsi, Republic of Korea; Korea Advanced Institute of Science and Technology (KAIST), Daejeon, Taejon-jikhalsi, Republic of Korea; Seoul National University Bundang Hospital, Seoungnam-si, Kyonggi-do, Republic of Korea

## Abstract

**Background:**

The adaptive immune system is known to be responsible for maintain long-term immune memory in an antigen-specific manner. However, recent studies have shown that in chronic infections such as CMV, HCV, and HIV, long-lasting modifications can occur in immune cells without antigen specificity. In the present study, we longitudinally investigated transcriptional characteristics of CD4^+^ T cells in extrapulmonary tuberculosis (EPTB) before, during and after anti-tuberculosis treatment.

**Methods:**

Peripheral blood mononuclear cells were isolated from patients with EPTB at three time points: before treatment (0 months), during treatment (2 months), and after treatment (6-8 months). Single-cell libraries were generated using Chromium Single Cell 3’ Library & Gel Bead Kit v3 (10X Genomics), and sequenced. The sequencing data were aligned to the reference human genome (GRCh38). The analysis was performed using R (v4.2.1).

**Results:**

We performed unsupervised clustering to classify a total of 49,317 cells into five major cell types, including T cells, B cells, NK cells, Monocytes, and Dendritic cells. Among 14,845 CD4^+^ T cells, we identified seven different subclusters according to highly variable genes. Comparison of the proportion of each CD4^+^ T cell cluster between healthy donors and patients with EPTB at different time points revealed an increased proportion of ‘Cluster 1’ in EPTB, even after anti-tuberculosis treatment. We calculated cluster-specific differentially expressed genes (DEGs) and found that ‘Cluster 1’ was characterized by up-regulation of *FOS, JUN, FOSB, JUNB, NFKBIA, NFKBIZ, SOCS3, SGK1, AREG*. Protein interaction network analysis of selected top 30 up-regulated genes in each cluster revealed the Activation protein 1 (AP-1) family genes were core genes of ‘Cluster 1’. Gene set enrichment analysis according to DEGs of CD4^+^ T cells revealed that AP-1 pathway gene sets in Pathway interaction database were down-regulated in healthy donor, while up-regulated in EPTB patients before, during, and after treatment.

Single-cell transcriptomes of PBMCs from extrapulmonary tuberculosis.

**Figure:**
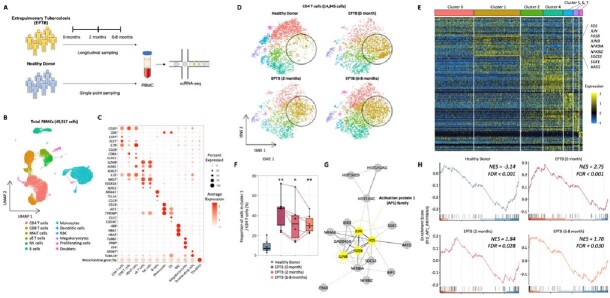

(A) Schematic representation of the experimental protocols. (B) Unbiased UMAP projection of 49,317 PBMCs colored by cell type information. (C) Dot plots showing the average normalized expression of marker genes in each cell cluster. (D) tSNE projection of 14,845 CD4+ T cells of healthy donor and patients with extrapulmonary tuberculosis. (E) Heatmap of cluster-specific differentially expressed genes for each CD4+ T cell cluster. (F) The proportion of cluster 1 cells in CD4+ T cells. (G) A network plot representing upregulated genes in cluster 1 CD4+ T cells. (H) Gene sets enrichment analysis of AP1 pathway in cluster 1 CD4+ T cells at different time points after anti-tuberculosis treatments.

**Conclusion:**

Our study revealed that CD4^+^ T cell transcriptomic changes associated with the AP-1 pathway persisted in patients with EPTB even after treatment. Further research is required to determine the clinical implication of these transcriptomic changes.

**Disclosures:**

**All Authors**: No reported disclosures

